# Psycho-Social Factors Associated with Intimacy Needs in Divorced and Widowed Older Chinese Women

**DOI:** 10.3390/ijerph191912360

**Published:** 2022-09-28

**Authors:** Zhe Ji, Elsie Yan

**Affiliations:** 1 Faculty of Social Sciences, Hong Kong Baptist University, Hong Kong 999077, China; 2 Department of Applied Social Sciences, The Hong Kong Polytechnic University, Hong Kong 999077, China

**Keywords:** sexuality and intimacy in older adults, active ageing, healthy ageing, older female, divorce, widowhood

## Abstract

The rapidly ageing population, the growing rate of divorce, and the longer life expectancy of women compared with men have resulted in a large proportion of older Chinese women being alone. The sexual health and intimacy needs of this group of women are ignored due to traditional Chinese cultural values that undermine older adults’ needs for sex and intimacy, and the subordinate position of women in society. The present study used a quantitative survey method to investigate bio-psycho-social factors associated with divorced and widowed older Chinese women’s sexual health and intimacy needs in two cities in Shanghai and Wuhan. The conceptual framework of this study was guided by cognitive stress theory, attachment theory, gender theory, socioemotional selectivity theory, objectification theory, and activity theory. We adopted a model involving demographic, biological, psychological, and social factors to unearth the mechanism influencing divorced and widowed older Chinese women’s sexual health and intimacy needs. Data were collected between October 2020 and March 2021 via face-to-face surveys. A convenience sample of 278 (N = 278) divorced and widowed older Chinese women was recruited (166 in Shanghai and 112 in Wuhan). Sexual health and intimacy needs were associated with current relationship status, financial status, physical health, attachment style, neuroticism, mental health, social support, prior marital conflict, and gender norm attitude. Sexual behaviour was associated with age, number of children, financial status, physical health, attachment style, neuroticism, prior marital conflict, gender norm attitude, sexual knowledge, sexual attitude, and intimacy attitude as predictors. Most participants in this study seemed to have found new intimate partners with whom to continue participating in sexual activities after their divorce or widowhood. The present study is one of the first examples of empirical research to examine the sexual health and intimacy needs of older Chinese women who are divorced or widowed. The findings generated by this study will inform the design and implementation of appropriate social programs for this demographic, and provide invaluable insight for social workers, educators, sex therapists, healthcare professionals, community workers, and policymakers working with this population.

## 1. Introduction

Ageing is a story of individual and family changes. It is also a story of loss—the loss of physical and mental function, the loss of family and friends, and the loss of one’s spouse. These losses occur at different rates for different individuals and groups; individuals and families often adapt to losses with changes in their behaviour or environment, making ageing a complex and dynamic process [1]. However, among older adults, family structures and broader social networks have undergone fundamental changes. Children leave home, retirement uproots individuals from their social networks at work, parents and friends pass away, and health problems begin to hinder social interactions [2]. During this period, one’s strongest source of support is often one’s spouse [1].

Market transition is a unique social process experienced by socialist countries in the process of transition from state-controlled economies to market-based economies [3]. In this large-scale economic reform, China is experiencing a major change in its population structure [4]. Population ageing refers to the increases in the number and proportion of older adults in society [5]. Since the 1990s, the process of population ageing in China has been accelerating. The People’s Republic of China, with a population of 1.41 billion by the end of 2020 [6], has the largest national population in the world. In 2010, there were 13.26% adults aged 60 years and over in China—2.93% higher than in the 2000 census [7]. By the end of 2019, the Chinese population aged 60 years and over had reached 197.99 million, or 18.13% of the total population [8]. By 2030, the population of adults aged 60 years and over is expected to grow by more than 50% [9]. By 2050, this will be almost one-third of the total Chinese population [10]. All these have resulted in China being one of the most rapidly ageing countries in the world [9].

On one hand, as a result of this rapidly ageing population, China is facing severe social and healthcare challenges [11]; on the other hand, the rapid economic development in China has led to improvements in health and medical care, which, in turn, lead to increased life expectancy [12]—this is more obvious in big cities. In big cities in China, such as Shanghai, Beijing, Shenzhen, Guangzhou, Wuhan, Chengdu, Tianjin, Xi’an, and Chongqing, people aged 60 years and above account for more than 10% of the total population [8]. As with the rest of the world, the life expectancy of women is generally higher than that of men in China. In 2010, women accounted for 60% of the population aged 80 years and above, and this is expected to continue to steadily increase in the future [13]. It is thus reasonable to state that the older population in China is a majority female one.

However, as the older population is increasing, a growing number of older adults are being confronted with singlehood, and the social structure in China is starting to change through divorce and widowhood [14]. In turn, this could lead to a decline in the level of family support for divorced and widowed older adults of lower income and poor health [15,16], most of whom require support both in terms of their finances and their emotions [17]. Even worse, compared with Western countries, China has more negative views on ageing, particularly when older adults are divorced and widowed [18].

Separation from previous partners does not take away the importance of sex and intimacy for individuals [19]. With remarriage and co-habiting becoming more common, relationships in later life are changing [20]. It is important at any age to express sexual and intimate needs, because doing so increases individuals’ awareness of themselves as valuable and respected human beings [21]. Continued sexual expression is associated with mental and physical well-being in later life [22]; sexual intimacy can reduce loneliness, contribute to a sense of comfort and well-being in older adults, and can also bring about a tremendous amount of pleasure [23].

Although they have positive impacts on the quality of life of older adults, the intimacy and sexuality of older adults is often misunderstood and ignored—many people are shocked by the idea that older adults remain sexually active over the age of 60 [24]. Despite the high prevalence rate of sexual problems (>50%), the frequency of sexual activity does not significantly decrease with age [25]; a considerable number of women and men engage in intercourse, oral sex, and masturbation, even in their 80s and 90s [26]. Sexuality contributes to perceived quality of life and well-being [27], and individuals in enduring intimate relationships tend to be satisfied emotionally and physically, including in terms of having relatively frequent sex [1]. Furthermore, some studies have indicated the diversity of older adults’ experiences and priorities, with both men and women discussing the importance of intimacy and bonding as an integral component of sex. In Western countries, dating is a common activity for older adults. In the United States, according to data from the 2005–2006 National Social Life, Health, and Aging Project, 14% of older divorced and widowed adults have dating-based relationships [14]. For women, the meaning of sex changes with age, with some repositioning the importance of sex, particularly penetrative intercourse [28]. Müller et al. [29] found that physical closeness, being in an intimate relationship, and the feeling of being “cared for” become more significant to older adults than sexual activity. Some research has suggested that sexual well-being and sexual activity are stronger among ageing men than ageing women [28], whereas other research [30] has shown that the relationship between sexual well-being and health is stronger in older women than among their male peers in a number of countries assessed worldwide. Furthermore, women have a survival advantage over men; the probability of female death is lower than that of male death at the same age, and is significantly lower than that of older men. As a result, older women are more likely to lose their spouse than men are [31]. This study, therefore, only recruited female participants.

The present study used a quantitative survey method to investigate bio-psycho-social factors associated with divorced and widowed older Chinese women’s sexual health and intimacy needs in two cities in China: Shanghai and Wuhan. The conceptual framework of this study is guided by cognitive stress theory, attachment theory, gender theory, socioemotional selectivity theory, objectification theory, and activity theory. The author adopted a model involving demographic, biological, psychological, and social factors to determine the mechanisms influencing divorced and widowed older Chinese women’s sexual health and intimacy needs. The present study results indicated that the participants had strong beliefs in egalitarian gender roles [32], high levels of body and sexual satisfaction [33], greater sexual knowledge, more permissive attitudes, and positive attitudes toward intimacy [34], as well as frequent engagement in sexual behaviour [34], and adult descendants formed an important part of the social support of the participants. In general, most of the research hypotheses were confirmed; however, no mediation effect of sexual health and intimacy needs was found between any of the factors and sexual behaviour. Most participants in this study seemed to have found new intimate partners with whom to continue participating in sexual activities after their divorce or widowhood. Most of the participants challenged and resisted the mainstream norms of intimate relationships and sexuality in their later years. Divorced and widowed older Chinese women are trying to open up possibilities for various intimate and sexual subjectivities in their later years by themselves.

The present study is one of the first examples of empirical research which examines the sexual health and intimacy needs of older Chinese women who are divorced or widowed. The first-hand data of this study have laid a foundation and provide a pathway for future quantitative research into the intimacy and sexuality of older Chinese women. The results from this study will help to improve our understanding of this issue, especially for social workers, healthcare providers, and policymakers working with bio-psycho-social factors associated with sexual health and intimacy needs among divorced and widowed urban older Chinese women. The findings generated by this study will also inform the design and implementation of appropriate social programmes for this demographic, and provide invaluable insight for social workers, educators, sex therapists, healthcare professionals, community workers, and policymakers working with this population.

## 2. Materials and Methods

### 2.1. Study Design and Settings

**Figure 1:**
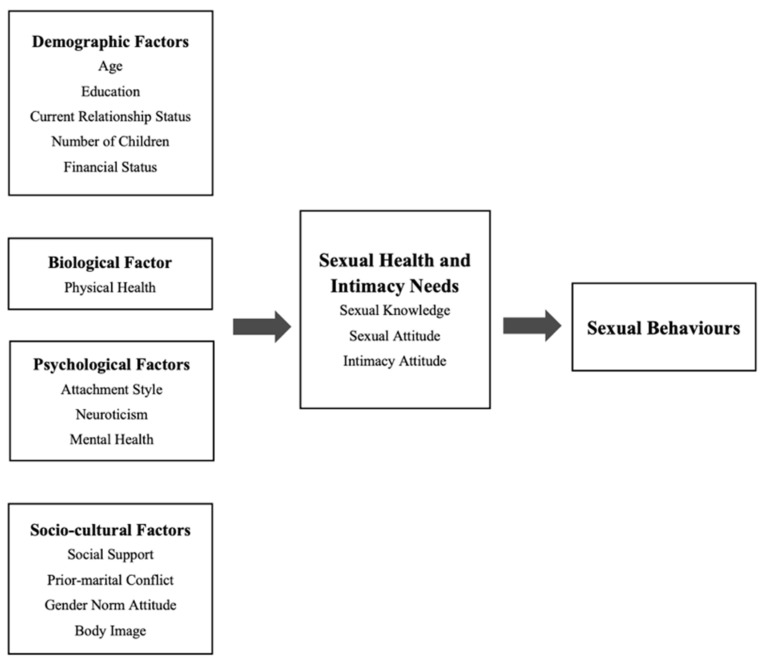
Conceptual framework.

In this study, a quantitative survey method was adopted to investigate the bio-psycho-social factors associated with divorced and widowed older Chinese women’s sexual health and intimacy needs in two cities in China: Shanghai and Wuhan. The cross-sectional survey in this study was conducted in six downtown districts in these two Chinese cities (three in each city) based on hypotheses regarding demographic, biological, psycho, and social variables (Figure 1).

### 2.2. Hypotheses

Demographic Variables:

**H1.**  *Age is negatively associated with current sex and intimacy needs.*

**H2.**  *Education level is positively associated with current sex and intimacy needs.*

**H3.**  *Current relationship status is positively associated with current sex and intimacy needs.*

**H4.**  *Number of children is negatively associated with current sex and intimacy needs.*

**H5.**  *Financial status is positively associated with current sex and intimacy needs.*

Biological Variable:

**H6.**  *Physical health is positively associated with current sex and intimacy needs.*

Psychological Variables:

**H7.**  *Attachment security is positively associated with current sex and intimacy needs.*

**H8.**  *Neuroticism is negatively associated with current sex and intimacy needs.*

**H9.**  *Mental health is positively associated with current sex and intimacy needs.*

Social Variables:

**H10.**  *Social support is positively associated with current sex and intimacy needs.*

**H11.**  *Prior marital conflict between ex-spouses is negatively associated with current sex and intimacy needs.*

**H12.**  *The belief in egalitarian gender roles is positively associated with current sex and intimacy needs.*

**H13.**  *Body image is positively associated with current sex and intimacy needs.*

### 2.3. Instruments

Financial status was measured using the InCharge Financial Distress/Financial Well-Being Scale (IFDFW; [35]). The IFDFW has been found to be a valid instrument in determining the financial status of respondents, and one that is highly correlated with the perception of financial well-being and accompanying stress (correlation equals well-being based on one’s personal financial condition and the level of stress) [36]. It has demonstrated an acceptable level of internal consistency, with a Cronbach’s alpha value of 0.93 [37]. Currently, there is no Chinese translation of this scale. Participants responded to each item on a 10-point Likert scale, with a higher total score indicating better financial well-being. A sample item of Financial Status is: How satisfied are you with your present financial situation?

Attachment style was measured using the Adult Attachment Scale (AAS; [38]). In previous studies, this scale has been found to be a valid instrument which is highly correlated with early attachment and adult intimacy experiences (correlation equals attachment style and attachment history) [38]. It has also demonstrated acceptable internal consistency, with a Cronbach’s alpha value ranging from 0.72 to 0.79 [39]. The scale has been translated and validated for Chinese populations, with an internal reliability of 0.795 [40]. Participants responded to each item on a five-point Likert scale, with a higher total score indicating more secure attachment. A sample item of Attachment Style is: I find it difficult to allow myself to depend on others.

Neuroticism was measured using the Neuroticism Facets of the Revised NEO Personality Inventory (NEO–PI–R; [41]). Previous studies have found it to be a valid instrument which is highly correlated with personality and smoking (correlation equals personality and behavioural tendencies) [42]. The NEO–PI–R has demonstrated high levels of internal consistency, with Cronbach’s alpha value of 0.92 for neuroticism, 0.89 for extraversion, 0.87 for openness to experience, 0.86 for agreeableness, and 0.90 for conscientiousness [42]. The scale has been translated and validated for Chinese populations, with an internal reliability of 0.63 [43]. Participants responded to each item on a five-point Likert scale, with a lower total score indicating a stronger personality. A sample item of Neuroticism is: I often worry about things that might go wrong.

Mental health was measured using the General Health Questionnaire (GHQ-12; [44]). Previous studies have found the GHQ-12 to be a valid instrument which is highly correlated with physical/psychological well-being and life quality (correlation equals mental health status and job-related factors) [45]. It has demonstrated acceptable internal consistency, with a Cronbach’s alpha value of 0.87 [46]. The scale has been translated and validated for Chinese populations, with an internal reliability of 0.84 [47]. Participants responded to each item on a four-point scale, with a higher total score indicating better psychological well-being. A sample item of Mental Health is: Have you recently been able to concentrate on what you’re doing?

Social support was measured using the Multidimensional Scale of Perceived Social Support (MSPSS; [48]). Previous studies have found the MSPSS to be a valid instrument which is highly correlated with perceived social support and anxiety (correlation equals perceived social support and depression) [48]. It has demonstrated acceptable internal consistency, with a Cronbach’s alpha value of 0.88 [49]. The scale has been translated and validated for Chinese populations, with an internal reliability of 0.89 [50]. Participants responded to each item on a seven-point scale, with a higher total score indicating stronger perceived social support. A sample item of Social Support is: There is a special person who is around when I am in need.

Prior marital conflict in an intimate relationship with an ex-partner was measured using Marital Conflict Scale (MCS; [51]). Previous studies have found the MCS to be a valid instrument which is highly correlated with marital satisfaction and marital conflict [52]. It has demonstrated acceptable internal consistency, with a Cronbach’s alpha value of 0.74 [52]. Currently, there is no Chinese translation of this scale. Participants responded to each item on a four-point scale, with a higher score indicating a higher level of prior marital conflict. A sample item of Prior marital Conflict is: How often do you and your prior husband have arguments about chores and responsibilities around the house?

Gender norm attitude was measured using the Gender Norm Attitude Scale (GNAS; [32]). Previous studies have found the GNAS to be a valid instrument which is highly correlated with gender and egalitarian beliefs [32]. It has demonstrated acceptable internal consistency, with a Cronbach’s alpha value of 0.67 for equity for women [53]. The scale has been translated and validated for a Chinese population, with an internal reliability of 0.77 [54]. Participants responded to each item on a two-point scale, with a higher total score indicating a stronger belief in egalitarian gender roles. A sample item of Gender Norm Attitude is: A good woman never questions her husband’s opinions, even if she is not sure she agrees with them.

Body image was measured using 28 items from the Sexual Adjustment and Body Image Scale (SABIS; [33]), based on the marital status and ageing of respondents. Previous studies have found the SABIS to be a valid instrument which is highly correlated with body satisfaction and sexual satisfaction (correlation equals effects of breast cancer on patients’ body image and their sexuality) [33]. It has demonstrated acceptable internal consistency, with a Cronbach’s alpha value of 0.71 [33]. The SABIS has been translated and validated for Chinese populations, with an internal reliability of 0.73 [55]. However, the 28-item scale constructed from the SABIS currently has no Chinese translation. Participants responded to each item on a five-point scale, with a higher total score indicating higher levels of body and sexual satisfaction. A sample item of Body Image is: Prior to being single, how satisfied were you with your physical attractiveness?

Sexual attitude and sexual knowledge were measured using both the attitude and knowledge questions of the Ageing Sexual Knowledge and Attitude Scale (ASKAS; [34]). Previous studies have found the ASKAS to be a valid instrument which is highly correlated with sexual cognition and ageing (correlation equals the general attitudes of older adults and their sexual behaviour) [56]. It has demonstrated acceptable internal consistency, with Cronbach’s alpha values ranging between 0.72 and 0.92 [57]. The scale has been translated and validated for Chinese populations, with an internal reliability of 0.85 [58]. In the present study, the true, false, and do not know questions measured individuals’ knowledge of sexuality, and the seven-point Likert scale measured people’s attitudes toward sexuality. A higher score indicates greater knowledge and more permissive attitude. A sample item of Sexual Attitude is: An aged person who shows sexual interest brings disgrace to himself/herself. A sample item of Sexual Knowledge is: Sexual activity in aged persons is often dangerous to their health.

Intimacy attitude was measured using the Intimacy Attitude Scale—Revised (IAS-R; [59]). Previous studies have found the IAS-R to be a valid instrument which is highly correlated with constructs of positive and negative intimacy within interpersonal relationships [60]. Correlation equals the components of intimacy attitude [61]. It has demonstrated acceptable internal consistency, with a Cronbach’s alpha value of 0.84 [59]. Currently, there is no Chinese translation of this scale. Participants responded to each item on a five-point Likert scale, with a higher total score indicating a more positive intimacy attitude. A sample item of Intimacy Attitude is: I am often anxious about my own acceptance in a close relationship.

Sexual behaviour was measured using the sexual activities subscale of the Senior Adult Sexuality Scale (SASS; [34]). Previous studies have found the SASS to be a valid instrument which is highly correlated with senior adults and their sexual interests, attitudes, and activities [56]. It has demonstrated acceptable internal consistency, with a Cronbach’s alpha value of 0.90 [34]. The scale has been translated and validated for Chinese populations, with an internal reliability of 0.88 [62]. Participants responded to each item on a six-point scale, with a higher total score indicating more frequent sexual behaviour. The time frame for measuring Sexual Behaviour was in the past year, the sample item of which is: How often do you initiate (start) sexual activity with your partner?

Information was also collected on participants’ age, education level (“never attended school”, “completed elementary school”, “some high school”, college graduate”, and “other”), current relationship status (“separation”, “divorced”, and “widowed”), and number of children. The question, “In general, how would you describe your health: poor, fair, good, very good, or excellent?” formed the physical health section of the survey.

### 2.4. General Procedures of Scale Translation and Adaptation

Applying Western scales to Chinese older adults may lead to confusion or misunderstanding, especially in terms of the appropriateness of the wording used. In the present study, three tools commonly used in Western society, the IFDFW, the MCS, and the IAS-R, were adapted into a form that was suitable for use in the Chinese context. In order to ensure the credibility of these questionnaires, three techniques were adopted: forward and back translation, suggestions from internal and external experts, and a pilot study.

#### Content Validity and Face Validity

The content validity and face validity were assessed in September 2020. The content validity of the questionnaire was assessed using item and scale content validity indices (I-CVI and S-CVI). The expert panels were asked to score the clarity, simplicity, and relevance of each question using a four-point Likert scale (ranging from 1: not relevant/unclear, to 4: completely relevant/clear). To calculate the I-CVI for each item, the proportion of experts who gave a rating of either 3 or 4 to the total number of experts was computed [63,64]. To measure the S-CVI, the average of all I-CVIs was calculated [65,66]. Content validity indexes were considered to be acceptable when values of I-CVI and S-CVI were at least 0.78 and 0.90, respectively [67].

Twenty older women in Wuhan qualitatively assessed the face validity of this survey. The inclusion criteria for pilot participants were as follows: must be a heterosexual female; must be aged 60 years or above; must be divorced or widowed; must have normal cognitive capability; must be able to understand and respond to the survey; and must be participating of their own volition. They were invited to answer two questions: (1) Do you think that this question and its response options are clear, concise, use correct grammar and are in an appropriate format?; and (2) Do you think that this question and its response options are relevant to sexual health and intimacy needs? All the pilot participants provided feedback in a 15 min telephone interview.

Finally, based on the feedback from experts and laypersons, a revised version of the questionnaire of present study was created.

### 2.5. Sampling Sites

Shanghai has the highest proportion of older adults and was the first city to experience an ageing population in China [68]. There were 305,200 older adults living alone as of the end of 2020 [69]. Wuhan is the capital of Hubei province. By 2021, those aged over 70 years old had reached 20.42% of the population [70].

### 2.6. Sample Size

It is necessary to accurately establish the minimal size of the sample so that it is representative of the entire population in the study area [71,72]. Generally, the larger the effect size, the stronger the statistical power. To determine regression sample sizes, Green [73] recommended a comprehensive formula—N ≥ 50 + 8m (where N is the sample size and m is the number of independent variables)—to test for multiple correlations.

Using this method of calculation, the adequate sample size for the present study was 178 (m = 16, 178 = 50 + 8 × 16). Taking into account factors such as incomplete questionnaires, heterogeneity of the sample composition, and attrition, the sample size was adjusted to 278 in order to obtain 178 valid questionnaires.

### 2.7. Participants

Due to the geographical location and administrative division of the participants, a multi-stage stratified sampling method was adopted. Six communities were chosen at random from every thirty communities in three downtown districts in Shanghai and Wuhan.

According to the Law of the People’s Republic of China on Protection of the Rights and Interests of the Elderly [74], in mainland China, the term “older adults” refers to adults over 60 years of age. Thus, in order to qualify for inclusion in this study, a participant:

(a) Had to be a heterosexual female;
(b) Had to be aged 60 years or above;
(c) Had to be divorced or widowed;
(d) Had to have normal cognitive capability;
(e) Had to be able to understand and respond to the survey;
(f) Had to be participating of their own volition.

Those who did not meet the inclusion criteria were not recruited. The confidentiality of the study was fully explained to the potential participants, with their written consent obtained before data collection. A small gift was given to every participant upon completion of the interview.

### 2.8. Data Collection

Data were collected between October 2020 and March 2021. Five local social workers in Shanghai and Wuhan were responsible for overseeing the survey in respective study sites. Two trained research assistants in each city were trained to administer the questionnaire should participants have difficulty reading or writing. Participants in Shanghai and Wuhan were referred by community workers from governmental and non-governmental organisations providing services for senior citizens. The aims and methods of this study were explained to all participants. Efforts were made to ensure that the participants were representative of different socio-economic statuses, reflecting the heterogeneity of the older female populations in Shanghai and Wuhan. Subjects were excluded if they refused to provide informed consent for the study. Most participants answered the questionnaires by themselves. 79 participants from Wuhan who had difficulty reading or writing and completed the questionnaires with assistance from the trained research assistants. On average, each questionnaire took 50 min to complete. Participants were given a bottle of hand sanitiser as souvenir upon completing the questionnaire.

Twelve questionnaires collected from Shanghai contains missing data and were excluded from the analysis. All personal identifiers were removed to ensure confidentiality. Hardcopies of questionnaires were destroyed once the data input was completed. The overall response rate for this study was 79%, yielding a final sample of 278 participants (166 from Shanghai and 112 from Wuhan).

In mainland China, obtaining data on the intimacy and sexual behaviour of older Chinese adults poses a challenge, because researchers in this field face resistance due to cultural and moral values, as well as shyness and other constraints. At this age, particularly for divorced and widowed older Chinese women, it is difficult to talk about sexual intimacy, because some of them may have experienced psychological trauma caused by divorce or widowhood, and the traditional beliefs in avoiding talking about sex, which may prevent them from disclosing personal information about themselves. Through our efforts and patience, and thanks to the support and cooperation of participants in this study, we successfully collected data from 278 divorced and widowed older Chinese women in Shanghai and Wuhan. Although the sample size of 278 was small, it was consistent with the actual situation in mainland China Mainland, which is also the larger sample size that can be collected in mainland China about this sensitive topic currently. In addition, the research assistants in the present study were trained over two sessions before data collection: they wrote down the answers provided by participants without contributing their own ideas. Therefore, there was little potential impact on the findings. All participants, local social workers, trained research assistants, and community workers in this study wore masks throughout the surveys, and the COVID-19 pandemic situation in Shanghai and Wuhan of China was well-controlled during the data collection period. Thus, the COVID-19 pandemic hardly affected data collection via face-to-face surveys in present study.

### 2.9. Data Analysis

Data analysis for the survey was carried out after all the questionnaires were completed. The analysis consisted of: (a) an assessment of the scale properties for internal consistency (Cronbach’s alpha); (b) analysis of the demographic characteristics of the sample participants for descriptive statistics, including means, standard deviations, and frequencies of age, education level, current relationship status, number of children, and financial status; (c) frequency of sexual behaviour; and (d) regression analysis for the model.

## 3. Results

All the analyses were performed using SPSS 26.0. The internal reliabilities of the major variables were good in the present study. The Cronbach’s alpha values were 0.98 for finance status, 0.80 for attachment style, 0.93 for neuroticism, 0.89 for mental health, 0.89 for social support, 0.89 for prior marital conflict, 0.94 for gender norm attitude, 0.89 for body image, 0.88 for sexual knowledge, 0.79 for sexual attitude, and 0.84 for intimacy attitude.

### 3.1. Demographic Factors

Descriptive statistical analyses were conducted on demographic characteristics of the participants, including city, age, education level, current relationship status, number of children, and financial status. Table 1 summarises the demographic characteristics of the sample participants. Of the 278 participants, 59.7% were from Shanghai and 40.3% were from Wuhan. The age of the participants ranged from 60 to 79 years old, with a median of 65.17 years old (SD = 4.52). Most had received a high school education (45.3%) and 10.1% had graduated from college. More than half (55%) were divorced, and 45% had lost their husbands. Most had one child (82.4%), and 12.6% had two children. Their financial statuses varied (SD = 16.48).

Table 2 presents the sexual behaviour frequency of the sample participants. The most frequent intercourse, sexual touch, oral sex, orgasm, use of sex objects, and sexual initiation all occurred once a week, then followed by one to two times a week.

### 3.2. Factors Associated with Sexual Health and Intimacy Needs

To determine the associations among various independent variables and intimacy needs, multiple regression analyses were conducted. The intimacy needs of the participants were used as the dependent variables. The demographic factors (age, education level, current relationship status, number of children, and financial status), biological factor (physical health), psychological factors (attachment style, neuroticism, and mental health), and socio-cultural factors (social support, prior marital conflict, gender norm attitude, and body image) were entered into the model as independent variables. In Model 1, the demographic factors were entered as control variables. Models 2 to 4 included the biological factor, psychological factors, and socio-cultural factors successively. The biological factor, psychological factors, and socio-cultural factors were transformed into standard scores before entering the regression model, to ensure that all scales were in the same metric. Multicollinearity was checked and ruled out when conducting the regression analysis. *p*-values smaller than 0.05 were deemed statistically significant. The results of the multiple regression analyses are shown in Table 3.

Four separate regression analyses were conducted to explore factors associated with intimacy attitude. In all these models, demographic factors were entered as control variables in Block 1 and independent variables concerned were entered in Blocks 2 to 4. Demographic factors alone accounted for 5% of the variance, with finance status positively associated with intimacy attitude. The biological factor explained 5% of the variance, with finance status significant in this model. Psychological factors explained 20% of the variance. Attachment style and mental health were significantly associated with intimacy attitude. Socio-cultural factors explained 26% of the variance in intimacy attitude, with social support and gender norm attitude associated with intimacy attitude.

### 3.3. Factors Associated with Sexual Behaviour

To determine the associations among various independent variables and general sexual behaviour, multiple regression analyses were conducted. General sexual behaviour was used as the dependent variable. The demographic factors (age, education level, current relationship status, number of children, and financial status), biological factor (physical health), psychological factors (attachment style, neuroticism, and mental health), socio-cultural factors (social support, prior marital conflict, gender norm attitude, and body image), and sexual health and intimacy needs (sexual knowledge, sexual attitude, and intimacy attitude) were entered into the model as independent variables. In Model 1, the demographic factors were entered as control variables. Models 2 to 5 included the biological factor, psychological factors, socio-cultural factors, and sexual health and intimacy needs successively. The biological factor, psychological factors, socio-cultural factors, and sexual health and intimacy needs were transformed into standard scores before entering the regression model, to ensure that all scales were in the same metric. Multicollinearity was checked and ruled out when conducting the regression analysis. *p*-values smaller than 0.05 were deemed statistically significant. The results of the multiple regression analyses are presented in Table 4.

Five separate regression analyses were conducted to explore factors associated with general sexual behaviour. In all of these models, the demographic factors were entered as control variables in Block 1 and the independent variables concerned were entered in Blocks 2 to 5. The demographic factors alone accounted for 24% of the variance, with financial status positively associated with sexual initiation. The biological factor explained 42% of the variance, with physical health significant in this model. The psychological factors explained 46% of the variance. Attachment style and neuroticism were significantly associated with sexual initiation. Socio-cultural factors explained 77% of the variance in sexual initiation, with prior marital conflict and gender norm attitude associated with sexual initiation. Sexual health and intimacy needs factors accounted for 37% of the variance, with age, financial status, sexual knowledge, sexual attitude, and intimacy attitude significantly associated with general sexual behaviour.

## 4. Discussion

### 4.1. Contributions

The present research is the first study on the intimate and sexual needs of divorced and widowed older women in China. This was a bold attempt, and the results of this study could contribute to eliminating the aspects of traditional Chinese culture which posit that divorced and widowed older women have no need for intimacy and are asexual.

Scale validation and adaptation procedures were rigorously implemented in this study. Suggestions and feedback from experts and participants were incorporated to ensure the reliability and validity of the instruments used. In the process, three important existing scales commonly used in sexuality research were revised or modified. The adapted scales, namely, the Financial Distress/Financial Well-Being Scale (IFDFW), the Marital Conflict Scale (MCS), and Intimacy Attitude Scale—Revised (IAS-R) proved to be psychometrically reliable when applied in the Chinese context. These adapted scales are suitable for future use in similar studies on, for instance, older adults living in rural China.

In the present study, a quantitative survey method was adopted to investigate bio-psycho-social factors associated with divorced and widowed older Chinese women’s sexual health and intimacy needs in two cities in China: Shanghai and Wuhan. The findings of the multiple regression analyses provide empirical support for the intervention applications of abundant predictors of sexual health and intimacy needs, as well as sexual behaviour. Most of the research hypotheses were confirmed; specifically, current relationship status, financial status, physical health, attachment style, neuroticism, mental health, social support, prior marital conflict, and gender norm attitude were predictors of sexual health and intimacy needs, whereas age, number of children, financial status, physical health, attachment style, neuroticism, prior marital conflict, gender norm attitude, sexual knowledge, sexual attitude, and intimacy attitude were predictors of sexual behaviour. However, no mediation effect of sexual health and intimacy needs was found between any of the factors and sexual behaviour. These predictors facilitate a better explanation of the bio-psycho-social factors of the sexual health and intimacy needs of divorced and widowed older Chinese women in Shanghai and Wuhan in China.

In addition to these predictors, we identified three significant findings of the present study. First, most participants showed greater sexual knowledge and more permissive sexual attitude, positive intimacy attitude, and frequently performed a variety of sexual behaviours, indicating their confidence and autonomy in regard to themselves and new forms of sexual intimacy. Second, most participants in this study seemed to have found new intimate partners with whom to continue participating in sexual activities after their divorce or widowhood, which is consistent with the idea that partnerships promote sexual activity, especially for older adults [75,76]. Third, children formed an important part of the social support of older women. This is a valuable finding that shows that, in mainland China, peer support for older adults may decrease with time, particularly for long-lived older women, and most of their social support comes from their children and family members. The findings of this study demonstrate that our understanding of intimacy and sexuality in older adults needs to both recognise and affirm diversity, while firmly recognising the continuing role of Chinese social norms and structures in shaping the sexual subjectivity of divorced and widowed older Chinese women. Moreover, most of the participants in this study challenged and resisted the mainstream norms of intimate relationships and sexuality in their later years, which suggests that divorced and widowed older Chinese women are trying to open up possibilities for various intimate and sexual subjectivities in their later years by themselves.

### 4.2. Implications for Practice

Continuous research on the intimacy needs and sexuality of divorced and widowed older women will help to eliminate Chinese social stereotypes about the intimacy and sexual behaviour of older adults, particularly those who are divorced and widowed, and to develop a culture in which older adults can comfortably and freely express their intimate and sexual needs without fear of ridicule or discrimination. The findings of this study could encourage older adults and their family numbers to understand the intimacy needs and sexuality of older adults, as well as the design and implementation of appropriate social programmes for this demographic, such as sexual health education, and community interventions and service projects.

The findings of this study further provide invaluable insight to social workers, educators, sex therapists, healthcare professionals, community workers, and policymakers working with this population. Specifically, social workers and community workers could organise more activities or groups for divorced and widowed older adults to increase their opportunities to find new intimate partners. Intervention programmes aiming to address the intimate and sexual needs of divorced and widowed older women could be implemented at the community level to cultivate more positive attitudes toward family members of divorced and widowed older women in regard to older adults’ intimacy and sexual behaviour. In addition, divorced and widowed older Chinese women with new partners should be encouraged to openly exchange their subjective feelings about their new intimate relationships and sexual lives, such as their sexual preferences, as well as their attitudes toward the changes in sexuality brought about by age. In China’s traditional gendered culture, some older women may hold conservative sexual attitudes. In this regard, intervention plans could be customised to challenge existing stereotypes and encourage divorced and widowed older Chinese women to regard sex as a permanent factor of life, as well as a part of human nature. The findings of this study highlight the importance of sexual health education for older women which, if widely implemented, could reduce the prevalence of AIDS in older adults; they should also have the opportunity to learn how to adapt to the sexual changes that come with ageing and find ways to express their sexual intimacy needs. Furthermore, it is necessary to improve the sexual health awareness of social workers, educators, sex therapists, healthcare professionals, and community workers interacting with this population.

### 4.3. Implications for This Research

This research represents the first empirical study in China to examine the sexuality and intimacy needs of older Chinese women who have experienced psychological trauma resulting from the breakdown of their marriages or the loss of their intimate partners. The present study used a quantitative survey method to investigate bio-psycho-social factors associated with divorced and widowed older Chinese women’s sexual health and intimacy needs in two cities in China: Shanghai and Wuhan. The conceptual framework of this study was guided by cognitive stress theory, attachment theory, gender theory, socioemotional selectivity theory, objectification theory, and activity theory. All scales in the present study were adapted for divorced and widowed older Chinese women for the first time. The use of these scales involved strictly implementing scale verification and adaptation procedures, incorporating the suggestions and feedback of experts and pilot study participants to ensure the reliability and validity of the tools used. In this process, four important scales commonly used in marriage and sexuality research were translated and modified: the InCharge Financial Distress/Financial Well-Being Scale (IFDFW), the Marital Conflict Scale (MCS), the Body Image Scale (SABIS), and the Intimacy Attitude Scale-Revised (IAS-R). In addition to the body image scale (SABIS), the three other metrics were found to be psychometrically reliable when applied in the Chinese context. These adjusted scales would be suitable for future similar studies, such as research involving older Chinese men or older adults in rural China.

In mainland China, obtaining data on the intimacy and sexual behaviour of older Chinese adults poses a challenge, because researchers in this field face resistance due to cultural and moral values, as well as shyness and other constraints. At this age, particularly for divorced and widowed older Chinese women, it is difficult to talk about sexual intimacy, because some of them may have experienced psychological trauma caused by divorce or widowhood, as well as the traditional beliefs in avoiding talking about sex, which may prevent them from disclosing personal information. Therefore, the findings and the first-hand data in this study have laid a foundation and provide encouragement for future research into the intimacy and sexuality of older Chinese women.

### 4.4. Directions for Future Studies

This study promotes the active ageing of older women in China. Future research could expand upon this study by looking at rural regions, older men and women who have never been married, divorced and widowed older heterosexual males, and queer individuals and other sexual minorities among older adults. In addition, a qualitative approach, or a qualitative/quantitative hybrid approach, could be explored in the future, which should lead to more comprehensive analyses in this area of research.

### 4.5. Limitations in the Present Study

There are several limitations to the present research. First, the sample in this study was not truly randomised; most of the participants in Shanghai and Wuhan were recommended by community workers, non-governmental organisations, and universities for older adults. Some older women who considered the research topic to be too sensitive refused to respond to the questionnaire, which may have affected the reliability of the research results to a certain extent. Second, the research design was cross-sectional; therefore, it is impossible to study the causality effects and longitudinal changes in the sexual heath and intimacy needs and practices of the participants. Third, the present study adopted self-reported data. When participants recalled their memories, biases and errors may have occurred. The information obtained may have been subject to recall bias or participants’ attention to social expectations. For instance, when asked about the current frequency of their sexual behaviour, the participants may have provided inaccurate information due to memory decline. No verification system has been established to verify responses provided in this manner. Fourth, some variables related to the theoretical model may have been ignored in this study. Further empirical evidence is needed to confirm the current framework for use in other regions and to explore other possible ways to better understand the framework used. Finally, the sample size of this study was relatively small, and the extent to which the findings represent divorced and widowed older urban Chinese women is unclear. In addition, in future studies, more efforts should be made to develop a specialised scale measuring the association between the body image and sexual behaviour of single older women.

## 5. Conclusions

The rapidly ageing population, the growing rate of divorce, and the longer life expectancy of women as compared with men have resulted in a large proportion of older Chinese women being alone. The sexual health and intimacy needs of this group of women are generally ignored due to traditional Chinese cultural values that undermine older adults’ needs for sex and intimacy, and due to the subordinate position of women in society. The present study used a quantitative survey method to investigate bio-psycho-social factors associated with 278 divorced and widowed older Chinese women’s sexual health and intimacy needs in two cities in China: Shanghai and Wuhan. We adopted a model involving demographic, biological, psychological, and social factors to determine the mechanisms influencing divorced and widowed older Chinese women’s sexual health and intimacy needs. The findings of this study suggest that most of the participants challenged and resisted the mainstream norms regarding intimate relationships and sexuality in their later years, which suggests that divorced and widowed older Chinese women are trying to open up possibilities for various intimate and sexual subjectivities in their later years. The findings of the present study could encourage older adults and their family members to understand the intimacy needs and sexuality of older adults, as well as the design and implementation of appropriate social programs for this demographic, such as sexual health education, and community interventions and service projects. This study further provides invaluable insights for social workers, sex therapists, healthcare professionals, community workers, and policymakers working with this population. In addition, the present study is one of the first examples of empirical research examining the sexual health and intimacy needs of older Chinese women who are divorced or widowed. The first-hand data of this study have laid a foundation and provide a pathway for future quantitative research into the intimacy and sexuality of older Chinese women. It is hoped that this research will encourage future studies on intimacy and sexuality among older adults in China.

## Figures and Tables

**Table 1:** Demographic characteristics of sample participants.

Characteristics		Participants *n* = 278
City (%)		
	Shanghai	59.7
	Wuhan	40.3
Age (mean ± SD)		65.17 ± 4.52
Education Level (%)		
	Never Attended School	0.7
	Primary School	18.0
	Junior Middle School	25.9
	High school	45.3
	College	10.1
Current Relationship Status (%)		
	Divorced	55.0
	Widowed	45.0
Number of Children (%)		
	Zero	0.4
	One	82.4
	Two	12.6
	Three	4.0
	Four	0.7
Finance Status (mean ± SD)		53.42 ± 16.48

**Table 2:** Frequency of sexual behaviour.

Sexual Behaviour	Frequency (%)					
	Not at All	Once per Month	Once Less Than One Month	Once a Week	Once or Twice per Week	Three Times or More per Week
Intercourse	12.9	0.4	5.0	54.7	21.2	1.4
Sexual Touch	12.9	0.7	6.5	43.5	31.7	0.4
Oral Sex	12.9	1.1	15.1	44.6	21.9	0
Orgasm	12.9	2.2	13.3	47.1	19.4	0.7
Use of Sex Objects	12.9	1.4	19.1	40.6	21.2	0.4
Sexual Initiation	12.9	1.4	14.4	40.6	25.9	0.4

**Table 3:** Multiple regression analyses for intimacy attitude.

Independent Variables	Dependent Variable: Intimacy Attitude			
	Model 1	Model 2	Model 3	Model 4
	Beta	Beta	Beta	Beta
Age	0.01	0.01	−0.01	0.06
Education Level	−0.02	−0.03	0.03	0.06
Current Relationship Status	0.03	0.03	0.04	0.03
Number of Children	−0.06	−0.06	−0.04	0.01
Finance Status	0.25 ***	0.24 ***	0.03	−0.04
Physical Health		0.06		
Attachment Style			0.28 ***	
Neuroticism			0.21 **	
Mental Health			0.20 *	
Social Support				0.51 ***
Prior marital Conflict				0.24 **
Gender Norm Attitude				0.23 *
Body Image				0.11
Model Statistics				
∆R Square	0.05	0.05	0.20	0.26
F	4.00	3.48	9.11	11.59
F Change	4.00	0.89	16.44	19.65

Note: Beta = Standardized Coefficients Beta. Statistically significant (* *p* < 0.05. ** *p* < 0.01. *** *p* < 0.001).

**Table 4:** Multiple regression analyses for general sexual behaviour.

Independent Variables	Dependent Variable: General Sexual Behaviour				
	Model 1	Model 2	Model 3	Model 4	Model 5
	Beta	Beta	Beta	Beta	Beta
Age	−0.19	−0.17	−0.18 *	−0.11	−0.25 **
Education Level	0.09	0.02	0.03	0.04	0.03
Current Relationship Status	0.10	0.08	0.04	0.05	0.03
Number of Children	−0.06	−0.02	−0.07	−0.04	−0.10
Finance Status	0.25 ***	0.11	0.09	−0.04	0.15 *
Physical Health		0.48 ***			
Attachment Style			0.32 ***		
Neuroticism			−0.37 ***		
Mental Health			0.00		
Social Support				−0.03	
Prior marital Conflict				−0.24 ***	
Gender Norm Attitude				0.65 ***	
Body Image				−0.04	
Sexual Knowledge					0.22 ***
Sexual Attitude					0.30 ***
Intimacy Attitude					0.26 ***
Model Statistics					
∆R Square	0.24	0.42	0.46	0.77	0.37
F	17.72	33.10	28.90	98.28	20.59
F Change	17.72	82.32	35.70	148.67	19.21

Note: Beta = Standardized Coefficients Beta. Statistically significant (* *p* < 0.05. ** *p* < 0.01. *** *p* < 0.001).

## Data Availability

Materials and anonymous data are available from the authors by request.
